# Combined computational analysis and cytology show limited depth osteogenic effect on bone defects in negative pressure wound therapy

**DOI:** 10.3389/fbioe.2023.1056707

**Published:** 2023-02-16

**Authors:** Xiu-Hong Huang, Li-Qin Zheng, Yue-Xing Dai, Shao-Nan Hu, Wan-Chen Ning, Si-Min Li, Yue-Guang Fan, Zi-Ling Lin, Shao-Hong Huang

**Affiliations:** ^1^ School of Stomatology, Stomatological Hospital, Southern Medical University, Guangzhou, China; ^2^ The First Clinical Medical College, Guangzhou University of Chinese Medicine, Guangzhou, China; ^3^ Department of Joint Surgery, First Affiliated Hospital of Guangzhou University of Chinese Medicine, Guangzhou, China; ^4^ Department of Orthopedic Trauma, First Affiliated Hospital of Guangzhou University of Chinese Medicine, Guangzhou, China

**Keywords:** negative pressure wound therapy (NPWT), computational fluid dynamics, CFD, bone marrow, bone mesenchymal stem cells (BMSCs), genes sequence, bone regeneration

## Abstract

**Background:** The treatment of bone defects remains a clinical challenge. The effect of negative pressure wound therapy (NPWT) on osteogenesis in bone defects has been recognized; however, bone marrow fluid dynamics under negative pressure (NP) remain unknown. In this study, we aimed to examine the marrow fluid mechanics within trabeculae by computational fluid dynamics (CFD), and to verify osteogenic gene expression, osteogenic differentiation to investigate the osteogenic depth under NP.

**Methods:** The human femoral head is scanned using micro-CT to segment the volume of interest (VOI) trabeculae. The VOI trabeculae CFD model simulating the bone marrow cavity is developed by combining the Hypermesh and ANSYS software. The effect of trabecular anisotropy is investigated, and bone regeneration effects are simulated under NP scales of −80, −120, −160, and −200 mmHg. The working distance (WD) is proposed to describe the suction depth of the NP. Finally, gene sequence analysis, cytological experiments including bone mesenchymal stem cells (BMSCs) proliferation and osteogenic differentiation are conducted after the BMSCs are cultured under the same NP scale.

**Results:** The pressure, shear stress on trabeculae, and marrow fluid velocity decrease exponentially with an increase in WD. The hydromechanics of fluid at any WD inside the marrow cavity can be theoretically quantified. The NP scale significantly affects the fluid properties, especially those fluid close to the NP source; however, the effect of the NP scale become marginal as WD deepens. Anisotropy of trabecular structure coupled with the anisotropic hydrodynamic behavior of bone marrow; An NP of −120 mmHg demonstrates the majority of bone formation-related genes, as well as the most effective proliferation and osteogenic differentiation of BMSCs compared to the other NP scales.

**Conclusion:** An NP of −120 mmHg may have the optimal activated ability to promote osteogenesis, but the effective WD may be limited to a certain depth. These findings help improve the understanding of fluid mechanisms behind NPWT in treating bone defects.

## Introduction

Open fractures occur when the broken bone is exposed to contaminations due to skin breach, significantly increasing complications (Louie, 2009). Open fractures often accompany severe bone defect, the reconstruction of segmental bone defects remains a challenge for surgeons, especially in cases of high energy injuries characterized by a high proportion of multi-tissue and infected defects (Murray et al., 2008; Mathieu et al., 2011; Hering et al., 2022; Mathieu et al., 2022).

Negative pressure wound therapy (NPWT) is a widespread surgical technique that promotes wound healing. The NPWT device creates a partial vacuum using suction, the effectiveness of which is explained by several main mechanisms of action, including vacuum draws of extracellular inflammatory fluid, blood, and debris; draws wound edges closer; and promotes granulation tissue formation (Sharp, 2013; Normandin et al., 2021). Evidence supports the positive results of NPWT in treating open fractures (Liu et al., 2018a; Kim and Lee, 2019; Grant-Freemantle et al., 2020; Qian et al., 2022), indicating that patients who received NPWT were less likely to have culture-positive wounds, develop clinical infections, and develop osteomyelitis. NPWT has been an effective method to heal soft tissue since 1993 (Fleischmann et al., 1993); however, its effects on bone regeneration have not been studied until recently (Zhang et al., 2013; Yang et al., 2014; Zhu et al., 2014).

Once a bone injury occurs, mesenchymal stem cells (MSCs) from various tissues (e.g., bone marrow, periosteum, vessel walls, muscle, and circulation) can migrate and differentiate into osteoblast lineage cells to participate in bone regeneration (Hadjiargyrou and O'Keefe, 2014; Garg et al., 2017). One study found that the osteogenic potential of MSCs can be regulated by mechanical stimulation (Ivanovska et al., 2015). Extrinsic mechanical cues and suitable stiffness can promote osteoblast differentiation *in vitro* and endochondral ossification of MSCs (Pelaez et al., 2012; Steward and Kelly, 2015). A few *in vitro* and *in vivo* studies have suggested that under suitable continuous or intermittent NPWT, bone regeneration may be accelerated by enhancing MSC proliferation, osteoblastic differentiation, and osteogenesis-related cytokine expression (Zhang et al., 2013; Yang et al., 2014; Zhu et al., 2021). Another study identified a novel autophagy axis by which negative pressure promotes osteoblast differentiation of MSCs and bone regeneration (Zhang et al., 2022).

As aforementioned, fluid flow in the bone marrow cavity caused by negative pressure (NP) is anticipated, confirming the osteogenic effect of NP; however, there is no study focusing on the fluid flow condition in the bone marrow cavity and investigating the effect of bone formation on fluid dynamics. The mechanics of the fluid and trabeculae in the marrow cavity under NP remain unknown. This challenge exists because we lack a direct method to detect fluid flow deep in the marrow cavity wrapped by the cortical bone and soft tissue. A realistic model for the interstitial fluid flow in the marrow cavity could help to understand how NPWT works on trabecular tissue and may optimize the protocols for NPWT on bone defects resulting from open fractures.

The computational fluid dynamics (CFD) method makes it possible to obtain a glimpse of fluid flow dynamics inside the marrow cavity during NPWT. CFD has been used to predict flow characteristics within the body, such as hemodynamics (Pandey et al., 2020; Kim et al., 2022; Roustaei et al., 2022). This study aims to conduct experimental measurements on human trabecular bone and develop a CFD model to investigate the effects of NP and NP-induced bone formation on fluid flow dynamics. We proposed *working distance* (WD) to quantify the dynamics effect of NP under various conditions. Furthermore, we conducted gene sequencing analysis and bone mesenchymal stem cells (BMSCs) osteogenic differentiation to verify the effect of different NP scales. The findings of this study can help improve our understanding of the physiological mechanisms underlying NPWT in treating bone defects.

## Material and Methods

### Sample preparation

An 83-year-old male who underwent right hip hemiarthroplasty due to a femoral neck fracture induced by a sideway fall was recruited. The femoral head was extracted during surgery and fixed with 4% paraformaldehyde for 48 h. The patient and family were notified and consented to contribute to the femoral head for research after hemiarthroplasty.

### Micro-CT scanning

**FIGURE 1:**
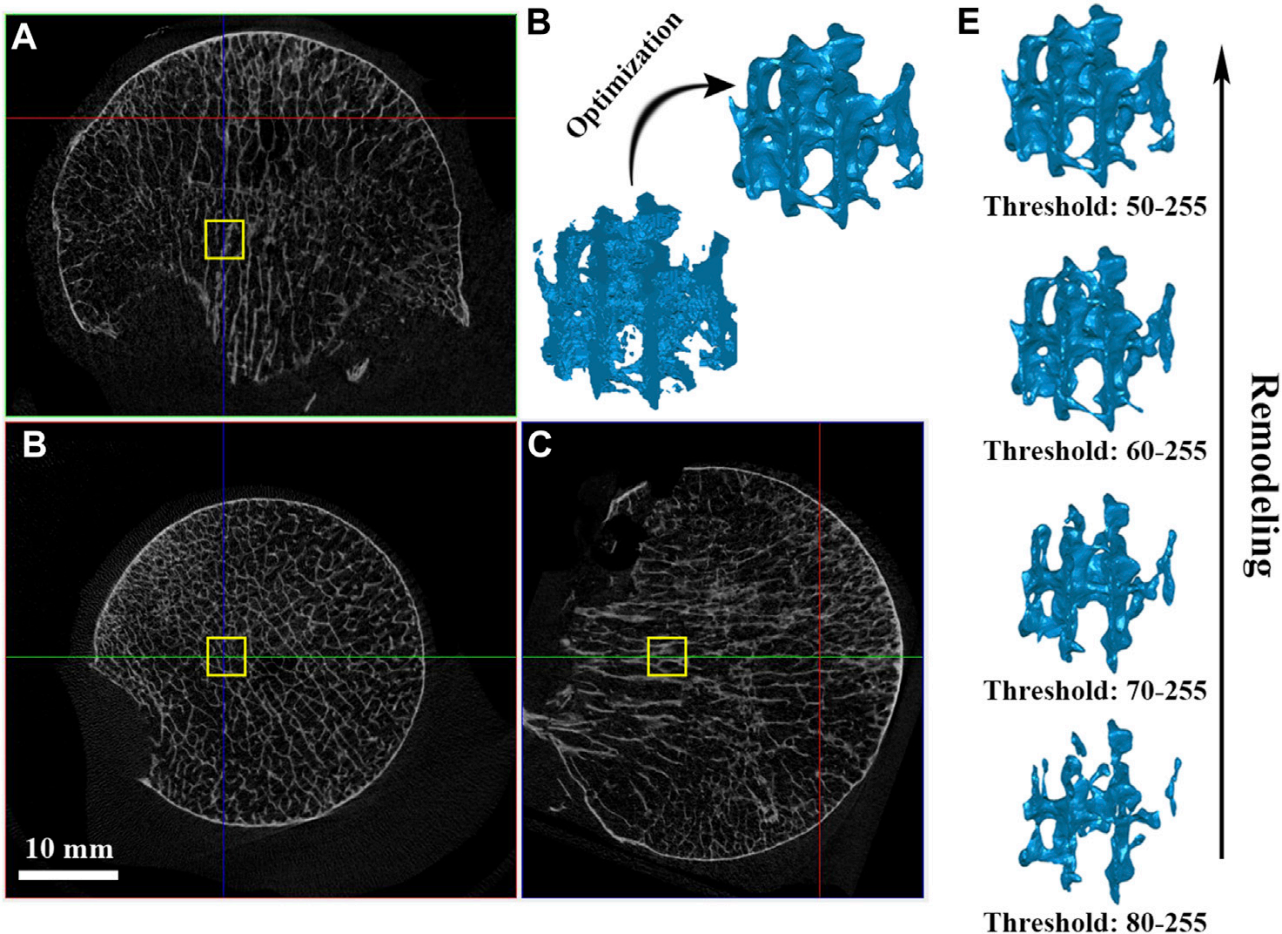
VOI trabeculae segmentation and simulating bone remodeling. **(A–C)** A cube of dimension 3 × 3 × 3 mm was taken as the volume of interest (VOI) from the domain of the femoral head; **(D)** Model optimization management; **(E)** Simulating individual trabecular bone remodeling by changing the global threshold.

The femoral head was scanned using a high-resolution micro-CT scanner (SkyScan 1276, Bruker, United States) at 42 μm resolution with 8-bit gray-level values. The x-ray source voltage was set to 100 kV, and the current was 200 μA. A rotation step as low as 0.8° was selected to obtain the finest resolution. Based on the micro-CT data, 8-bit bitmaps (BMP) of every cross-section along the femoral neck axis were reconstructed using the bundled software Nrecon 1.7.1 (Bruker, United States). DataViewer 1.5.6 (Bruker, United States) software was employed to orient the cross-sectional images perpendicular to the main compressive trabecular bone in the femoral head. Finally, in CTAn1.16.8 (Bruker, United States) software, a global threshold of 50–255 was chosen to segment the trabecular bone on the BMP images, and a cube with dimensions of 3 × 3 × 3 mm was taken as the volume of interest (VOI) from the domain of the femoral head, as shown in Figures 1A, C.

The VOI cube was first used to investigate the anisotropy effect by applying NP in different directions. Further, to investigate the effect of individual trabecular structure remodeling on fluid dynamics, a cohort with a low global threshold limit from 80 to 50 at an interval of 10 was chosen to segment out trabeculae under the same VOI, and their bone volume fraction (BV/TV) was recorded (Figure 1E). VOI trabeculae demonstrate higher BV/TV with a lower global threshold limit setting; therefore, the effect of using a different lower threshold when segmenting trabecular bone can be assessed (Muller and Ruegsegger, 1996; Barak and Black, 2018). The VOI trabeculae were saved as “stl” files for subsequent model optimization.

### Three-dimensional model optimization

Since the raw “stl” VOI trabeculae derived from CTAn software were full of noises (e.g., tiny spikes, unconnected elements, and unclosed pores) and were too rough for computational modeling, some polishing steps were undertaken using Geomagic studio 2013 software (GEOMAGIC Inc., United States). Optimization includes the “Mesh Doctor” (to detect and fix kind of flaws automatically) and “Remesh” (to acquire uniformly distributed two-dimensional elements). They were carefully operated to maintain the trabecular structure (Figure 1D). Simultaneously, a group of element sizes was set for the convergence test before the formal experiments. After optimization management, the VOI trabeculae were saved as “stl” files again, ready for the CFD model establishment.

### CFD establishment and simulation

**FIGURE 2:**
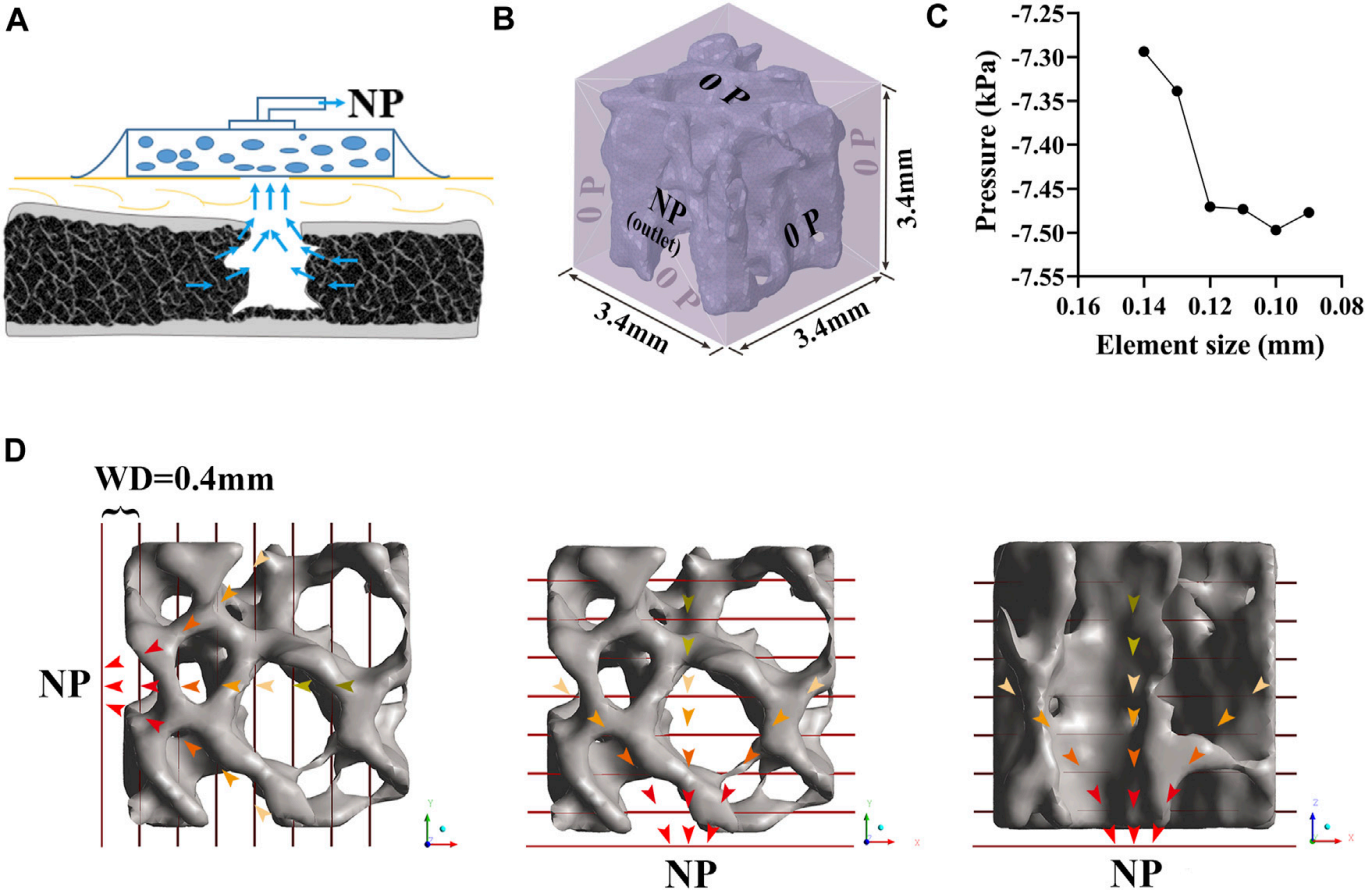
Development of CFD model of trabeculae. **(A)** Bone defect treated by NPWT. **(B)** Fluid domain and boundary condition. A 3.4 × 3.4 × 3.4 mm fluid box represents the marrow region; one of the planes was assigned as an outlet with NP while the others were inlets with zero pressure; the trabecular bone was considered rigid and had no slip, and the simulated marrow was assumed to be a steady-state, laminar, and incompressible Newtonian fluid. **(C)** Convergence study for accuracy of the CFD simulation. **(D)** NP was applied in three directions (along the X-, Y-, and Z-axis, respectively). A series of the cross-sectional plane was placed at an interval of 0.4 mm, representing the WD from NP sources. Each plane’s contents (including pressure, velocity, and shear stress) were expressed as median (25th–75th percentile).

In the SpaceClaim module of ANSYS 19.0 (Ansys Inc., United States), the polished VOI trabeculae were enclosed by a 3.4 × 3.4 × 3.4 mm fluid box to represent the marrow region. Then, the fluid-solid surface models were volumetric-meshed as tetrahedral elements using Hypermesh software (version 14.0; Altair Inc., United States) and assigned to the outlet and inlet planes. In each CFD simulation, the fluid box was assigned one outlet with NP and the other five planes were set to the inlet with zero pressure (0 P), simulating the bone marrow environment under a standard atmospheric pressure (Figures 2A, B). Although there are some differences between various NPWT systems concerning the filler material used (foam vs. non-adherent antimicrobial gauze) and the connecting suction catheter (integrated with a pressure sensor vs. flat drain), the intensity of NP ranges from −50 to −200 mmHg in most clinical practice (1 mmHg = 133 Pa) (Normandin et al., 2021), depending on the wound condition (Meloni et al., 2015; Agarwal et al., 2019). In the present study, NPs of −80, −120, −160, and −200 mmHg were applied along three principal directions (along the X-, Y-, and Z-axis, respectively) for anisotropy investigation. To study the effect of trabecular structural remodeling on fluid dynamics, an NP of −80 mmHg was chosen as a representative (Costa et al., 2020; Shiels et al., 2021).

The trabecular bone was considered rigid and had no slip wall, and the simulated marrow was assumed to be a steady-state, laminar, and incompressible Newtonian fluid with a density of 1.06 g/cm^3^ and viscosity of 0.4 Pa∙s (Rabiatul et al., 2021). The Navier-Stokes equation was adopted to define the flow behavior of viscous fluids. However, the effect of gravity was neglected. An iterative method was used to solve the equation for a steady flow, and convergence was identified when the relative tolerance was less than 0.001. Our preliminary study suggested that an element size of 0.10 mm of the fluid zone and trabeculae is sufficient to converge a steady fluid flow solution (Figure 2C).

As shown in Figure 2D, we defined the WD to enable post-processing of the CFD simulation. Specifically, a series of cross-sectional planes were placed at an interval of 0.4 mm to represent the WD from NP sources to the distal region. Cross-sectional planes were placed perpendicular to the NP direction. The resultant contents of each plane, including the pressure and shear stress on the bone and the velocity of marrow, were expressed as the median (25th–75th percentile) and reported as mean and standard deviation (‾x ± s) for regression (Wu et al., 2021).

### *In vitro* NP treatment

The pressure, shear stress on the bone, and the velocity of marrow were determined using CFD simulation, we then attempted to verify the effect of different scales of NP on BMSCs gene expression, proliferation and osteogenic differentiation. By combining CFD results and cytological experiment, we could speculate how NPWT works within the bone tissue.

**FIGURE 3:**
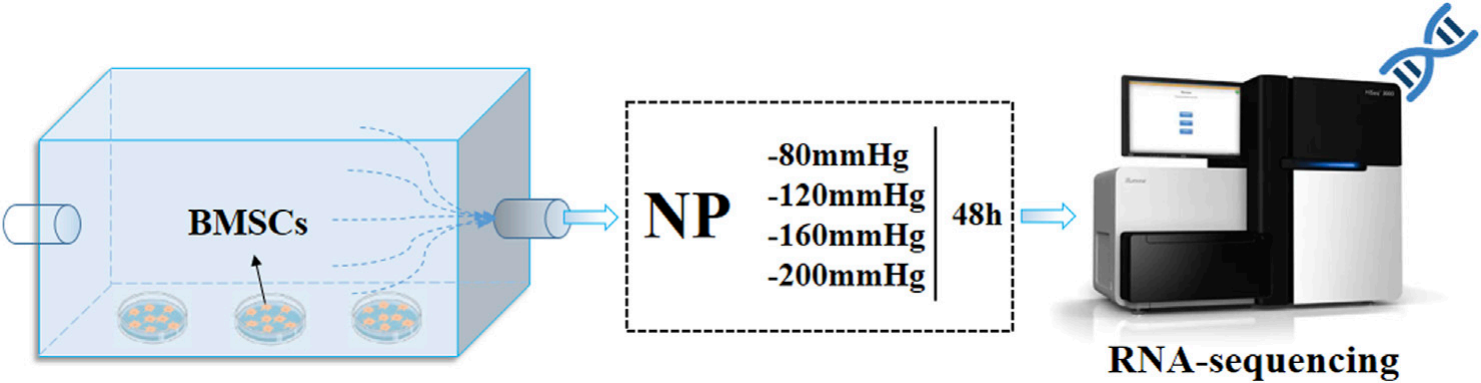
BMSCs culture under NPs and RNA-sequence analysis. BMSCs were cultured under four groups of NPs for 2 h per day, total 2 days, then follow the RNA-sequence analysis.

As shown in Figure 3, mouse BMSCs were seeded separately in a 24-well plate at a density of 1×10^4^ cells per well and cultured for 24 h. After that, the plates were placed in cell incubator with air inlet channel sealed and outlet channel linked to negative pressure device. The NP was controlled and adjusted using a vacuum pump (VSD Medical Technology Co., Ltd., customized, China) with a pressure sensor. The applied pressures of NP treatment were −80 mmHg, −120 mmHg, −160 mmHg, and −200 mmHg with a continuous pattern of 2 h per day, total 2 days for each plate.

### RNA-sequence analysis

After NP treatment, BMSCs were collected and harvested, and total RNA was prepared using the GenCatch TM Total RNA Extraction Kit (Epoch Life Sciences, 1,660,050) for RNA-sequence analysis following the manufacturer’s instructions (Illumina, Hiseq 2500, United States). Sequencing reads were mapped to the rat genome rn6 using HISAT2, and tag counts were summarized at the gene level using SAMtools, which allowed only one read per position per length. Differentially expressed genes (DEGs) were analyzed using edgeR. Gene set enrichment analysis, including Gene Ontology (GO) and Kyoto Encyclopedia of Genes and Genomes (KEGG), was performed using DEGs with clusterProfiler. GO analysis was visualized using topGO, and Fisher’s exact test was used for statistical analysis. KEGG analysis was performed using clusterProfiler, and the hypergeometric test was used for statistical analysis.

### Cell viability of BMSCs

After NP treatment, cells were continue to cultivate. At day 1, 3 and 7, the different medium was removed and replaced by 1 mL medium containing 100 μL cell counting kit-8 (CCK8) solution for 2 h incubation at 37 °C. After that, the optical density (OD) values of the cells in different medium were measured at 450 nm using an enzyme-linked immunosorbent assay plate reader (Multiskan MK3, Thermo Electron Corporation, United States).

### Alkaline phosphatase activity (ALP) of BMSCs

BMSCs cells were seeded in 24-well culture plates as described in section 2.5. After 7 days of culture in different NP (2 × 10^4^ cells/well), the qualitative and quantitative ALP activity of BMSCs were characterized using BCIP/NBT alkaline phosphatase color development kit (Beyotime, China) and alkaline phosphatase kit (Nanjing Jiancheng, China), respectively.

For the qualitative ALP activity, the cells were fixed in 4% paraformaldehyde for 15 min and gently rinsed with PBS. The ALP activity staining was performed by a BCIP/NBT Alkaline Phosphatase color development kit according to the manufacturer’s instructions. Images were recorded with an Olympus MVX10 MacroView (Japan). For quantitative analyses of ALP activity, cells were incubated in RIPA lysis Buffer for 60 min. Before the analysis of ALP activity, total protein concentration (mg/mL) and ALP activity (units/mL) were measured following the manufacturer’s instructions with an enhanced BCA protein assay kit (Beyotime, China) and ALP reagent kit, respectively. ALP levels were normalized to the total protein content.

### Mineralized matrix formation

12 days after culturing under NP, the extracellular matrix mineralization of BMSCs were evaluated by the alizarin red S staining and calcium contents. The BMSCs were fixed in 4% paraformaldehyde for 15 min and gently rinsed with PBS. Finally, the sample were then incubated with 40 mM alizarin red S (ARS) staining solution (Sigma) for 30 min at room temperature. Images were recorded with an Olympus MVX10 MacroView (Japan). For quantitative analysis, calcium contents were measured using a calcium colorimetric assay kit (Nanjing Jiancheng, China) according to the manufacturer’s instructions. All samples were examined at less three times.

### Statistical analysis

The pressure, shear stress upon the trabeculae, and flow velocity of the simulated marrow were analyzed. Specifically, these dependent variables were estimated as a function of the distance to NP sources (i.e., WD). Before regression analysis, we conducted a curve-fitting exam using SPSS software (version 20.0; IBM Inc., United States) and found a better-matched exponential function between WD and the dependent variables compared to other potential functions; therefore, a non-linear, least squares regression was used to determine the coefficients of an exponential function in the form:

$$Y=A\cdot e^{Bx},$$

where *A* and *B* are coefficients, *e* is the natural constant (Euler number), and *Y* is one of the three observed dependent variables estimated as a function of WD (*x*). The resulting coefficients with a *p*-value and correlation coefficient *R* ^*2*^ are included.

Cytological experiment results are presented as the mean ± standard deviation (SD). Statistical comparisons were performed using a one-way analysis of variance followed by a Bonferroni test for multiple comparisons. Values of *p* < 0.05 were considered statistically significant.

## Results

### Anisotropy investigation

**FIGURE 4:**
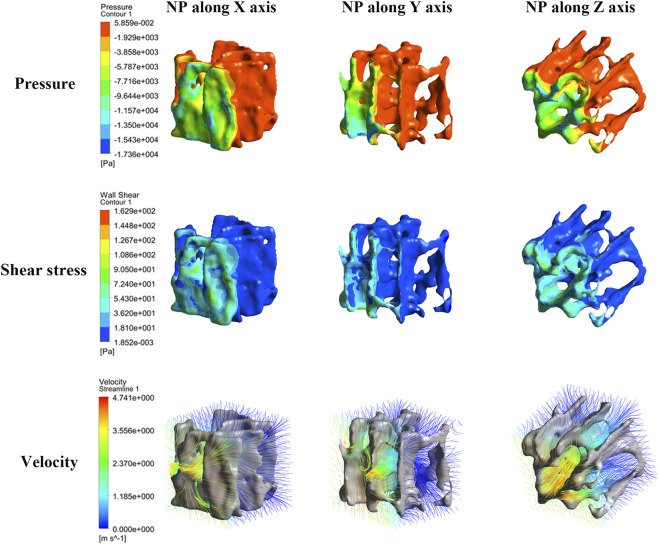
Nephogram of dependence variables induced by NP along the X-, Y-, and Z-axis (consider NP −80 mmHg for clarity). The pressure, shear stress and morrow flow velocity concentrates near to the NP source.

As Figure 4 shows, the closer the NP source, the higher the pressure and shear stress on the trabeculae and the fluid velocity. Pressure and shear stress share a similar concentration area; however, the maximum shear stress tends to be located at tiny trabeculae. The fluid flow had significant anisotropy under NP in different directions; when NP was along the mechanical axis (Z-axis) of the trabeculae, the fluid flow was steady and laminar. A turbulent streamline can be found near the NP source when the NP is along the off-mechanical axis (non-Z-axis) of the trabeculae.

**FIGURE 5:**
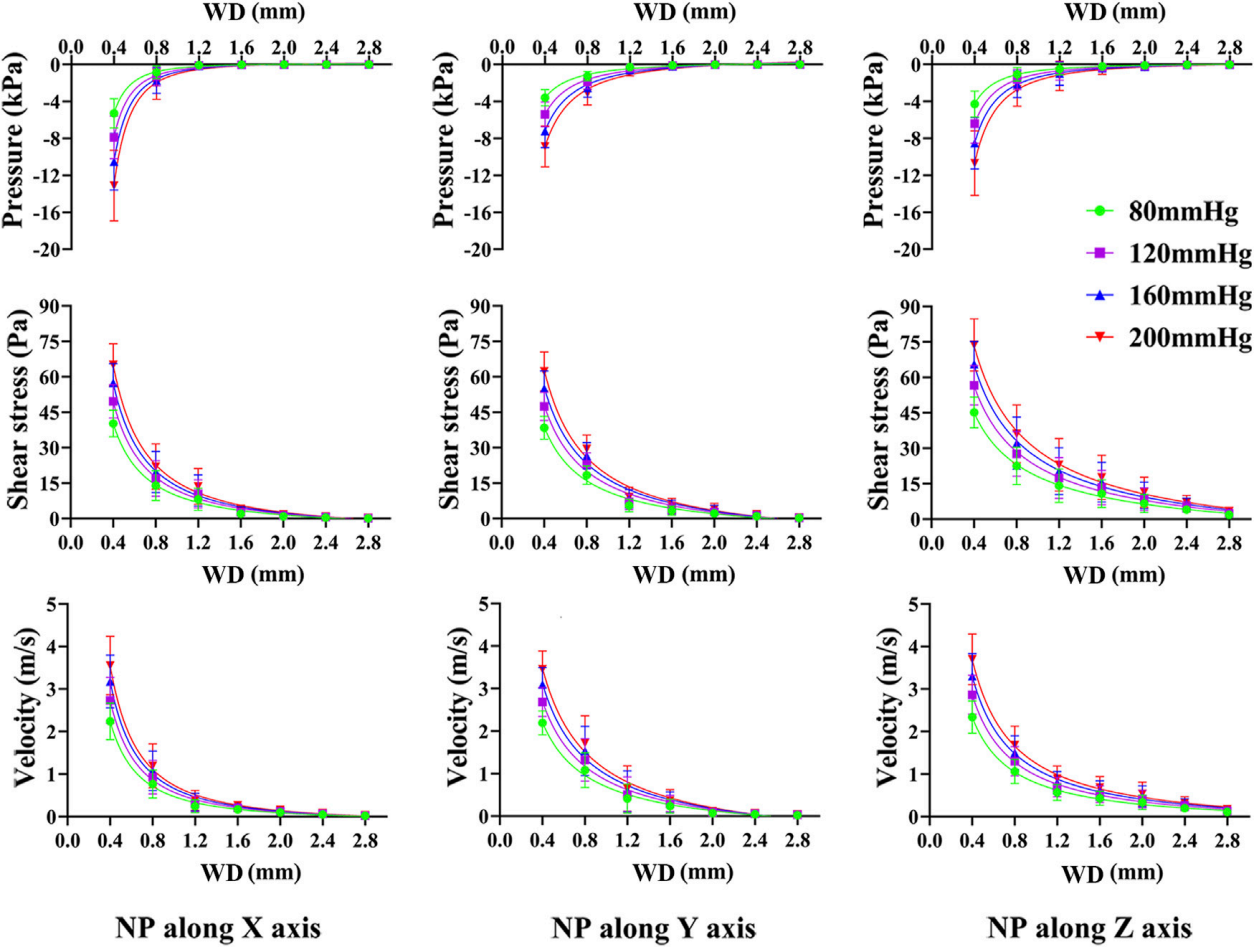
Non-linear regression curves between dependent variables and WD under various NP scales and directions. The pressure, shear stress on trabeculae and marrow fluid velocity decrease exponentially with an increase in WD. NP induce great difference in pressure, shear stress and marrow fluid velocity only within a short WD.

Pressure, shear stress, and velocity shifted down significantly from the NP source to 0.4 mm and 0.8 mm WD planes, especially when the NP was along the off-mechanical axis. The average median pressure, shear stress, and velocity at each WD for different NP are shown in Figure 5. All dependent variables at each WD plane increased with NP, and the increment of the first two WD planes was still more significant than that of the others. The changing tendency seems to be steadier when NP is along the mechanical axis than along the off-mechanical axis, indicating the influence of anisotropy of trabeculae on fluid dynamics. Although there was a 2-fold increase in NP (from 80 to 160 mmHg), there was no corresponding 2-fold increase in the dependent variables.

From Supplementary Tables S1–S4, non-linear regression demonstrated an exponential expression with a high confidence coefficient (R^2^ > 0.96) between dependent variables and WD, regardless of the NP direction and scale. The pressure, shear stress on the trabeculae, and fluid velocity were well defined by the WD.

### Effect of trabecular structure remodeling

We used an NP of −80 mmHg as an example to investigate the effect of trabecular structure remodeling on fluid dynamics because the results derived from other scales of NP would be found to be similar to the −80 mmHg induced outcomes.

**FIGURE 6:**
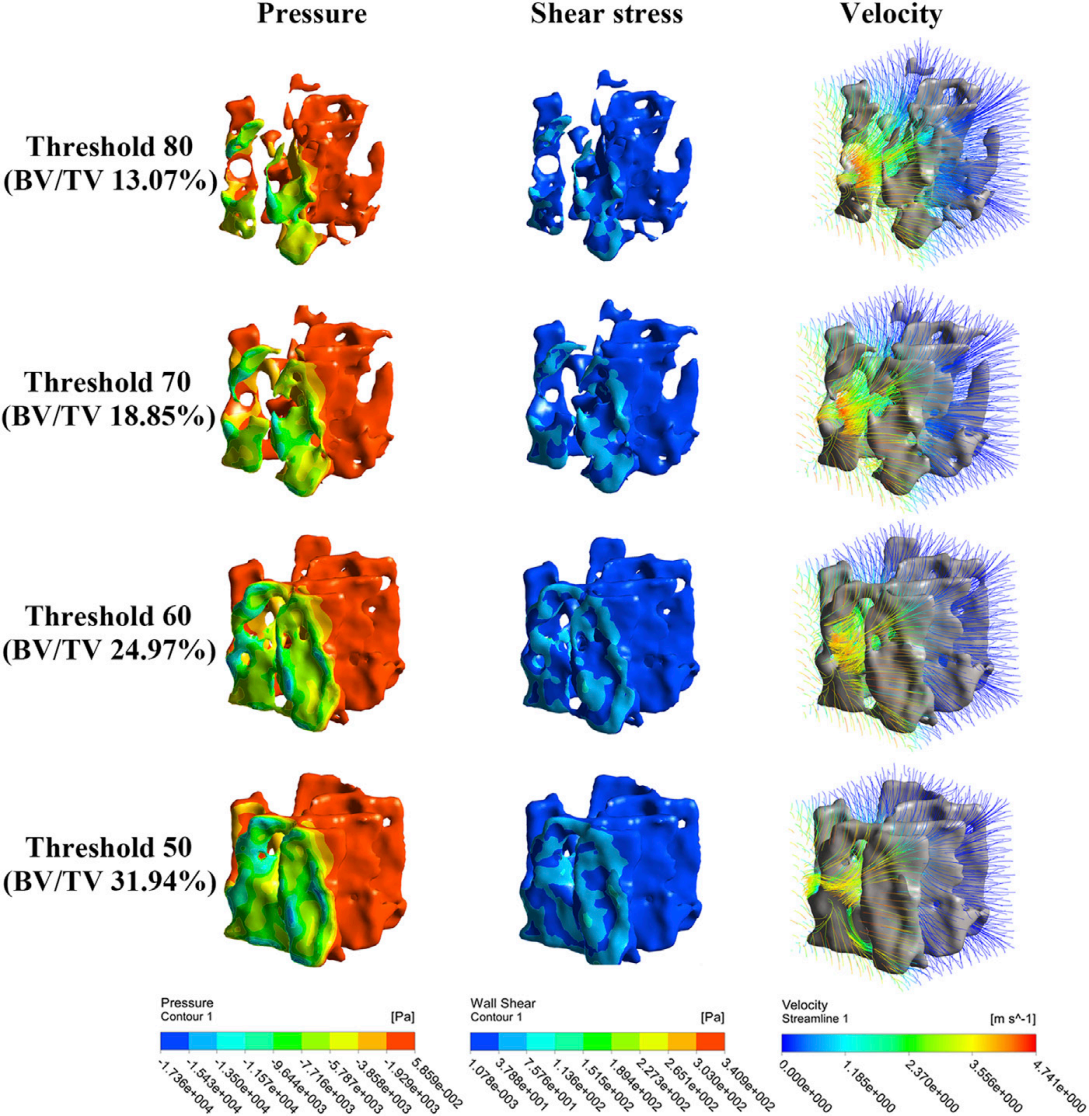
Nephogram of dependence variables result from simulated bone remodeling (take NP along the X-axis for clarity). The pressure, shear stress and morrow flow velocity concentrate near to the NP source.

Figure 6 shows that the concentrations of the pressure and shear stress are located near the NP source. With trabecular remodeling, the concentration area increases accordingly, but the concentration depth does not change significantly. Streamlines become sparse and turbulent, particularly in regions close to the NP source.

**FIGURE 7:**
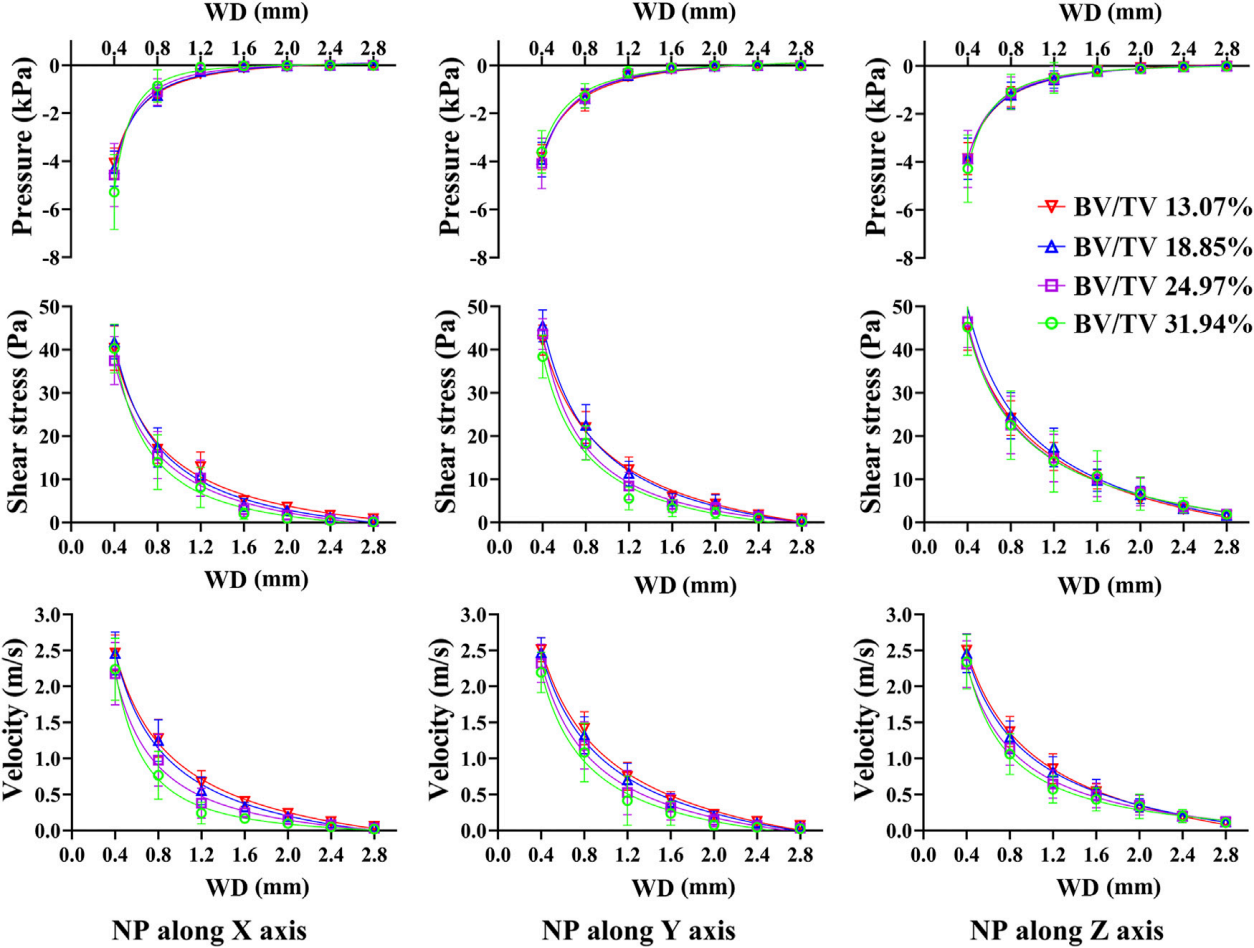
Non-linear regression curves between dependent variables and WD under different structure remodeling levels. The pressure, shear stress on trabeculae and marrow fluid velocity decrease exponentially with an increase in WD. As bone remodeling, the pressure, shear stress on trabeculae and the morrow flow velocity decrease significantly only with a short WD.

As Figure 7 shows, pressure, shear stress, and velocity shifted down significantly from the NP source to 0.4 mm and 0.8 mm WD planes, especially when NP is along the off-mechanical axis, which is similar to the aforementioned results. Pressure, shear stress, and velocity decrease at most WD planes with structural remodeling, except at the 0.4 mm WD plane. This decreasing trend was more evident in the shear stress and velocity when the NP was along the off-mechanical axis. There was an approximately 2-fold increase in BV/TV (from 13.07% to 24.97%, corresponding to global threshold limit of 80 and 60); however, the decrease in the dependent variables was not 2-fold accordingly.

Nonlinear regression also demonstrated an exponential expression with a high confidence coefficient (R^2^ > 0.97) between the dependent variables and WD. The confidence coefficient tended to decrease with structural remodeling. The pressure, shear stress, and velocity were better defined by the WD on the resorbed trabecular bone (Supplementary Tables S5–S8).

### Effects of NP on BMSCs genes expression

**FIGURE 8:**
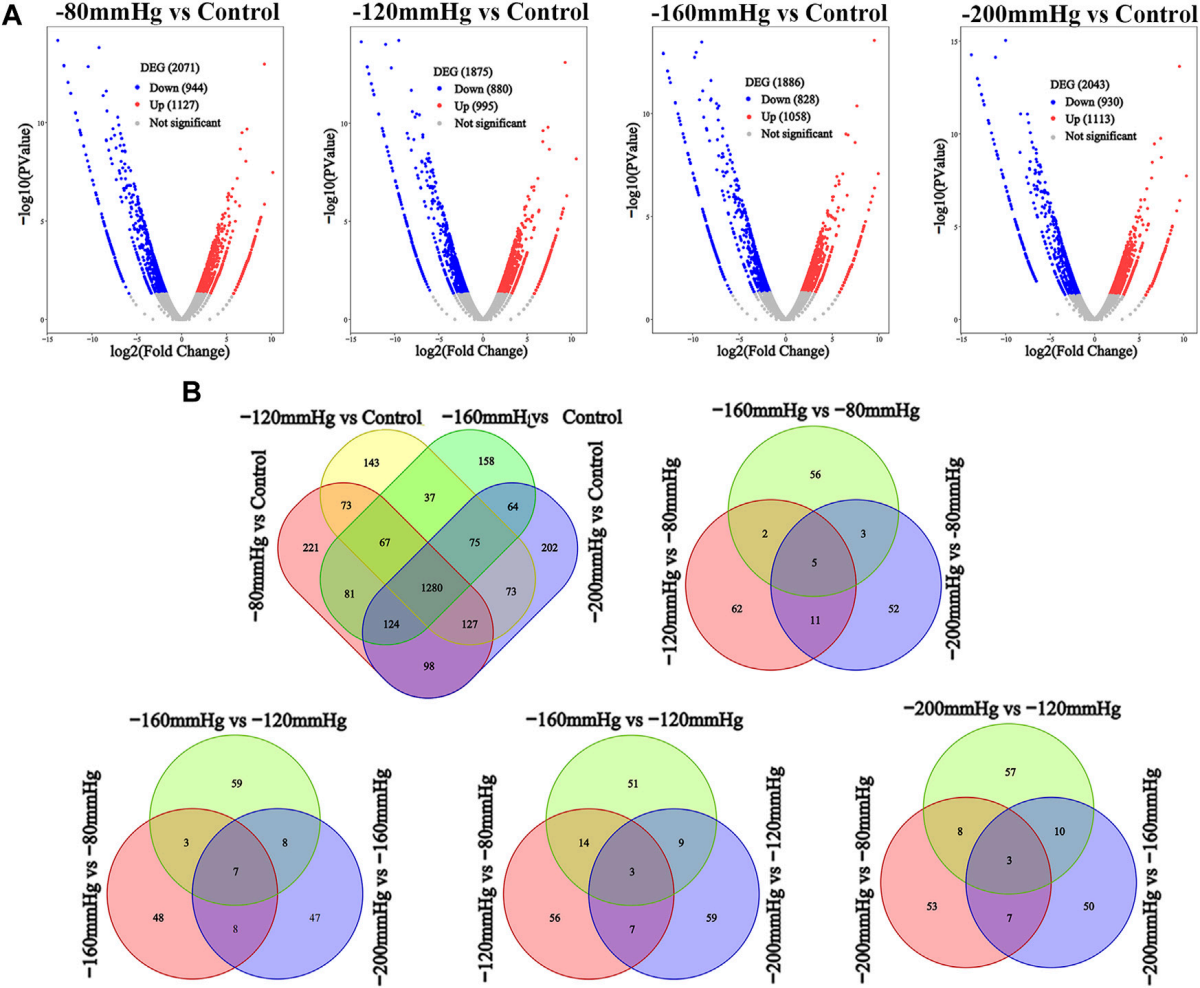
DEGs analysis among four NPs vs. control. **(A)** Volcano analysis of DEGs in −80 mmHg vs. control, −120 mmHg vs. control, −160 mmHg vs. control and −200 mmHg vs. control, respectively. **(B)** Venn diagram of the DEGs in BMSCs with NP treatment.

We performed RNA sequence analysis to verify the effect of different NP scales on BMSCs gene expression. Volcano analysis (Figure 8A) identified 2071 DEGs after −80 mmHg treatment, of which 1127 genes were upregulated, and 944 genes were downregulated. There were 1875 DEGs after −120 mmHg treatment, with 995 upregulated and 880 downregulated genes. For −160 mmHg treatment, volcano analysis identified 1886 DEGs, which included 1058 upregulated genes and 828 downregulated genes. In addition, 2043 DEGs were identified in the −200 mmHg group, of which 1113 genes were upregulated, and 930 genes were downregulated. From the Venn diagram (Figure 8B) of the −80, −120, −160, and −200 mmHg NP groups, more DEGs were observed in the −120 mmHg group than in the other groups.

**FIGURE 9:**
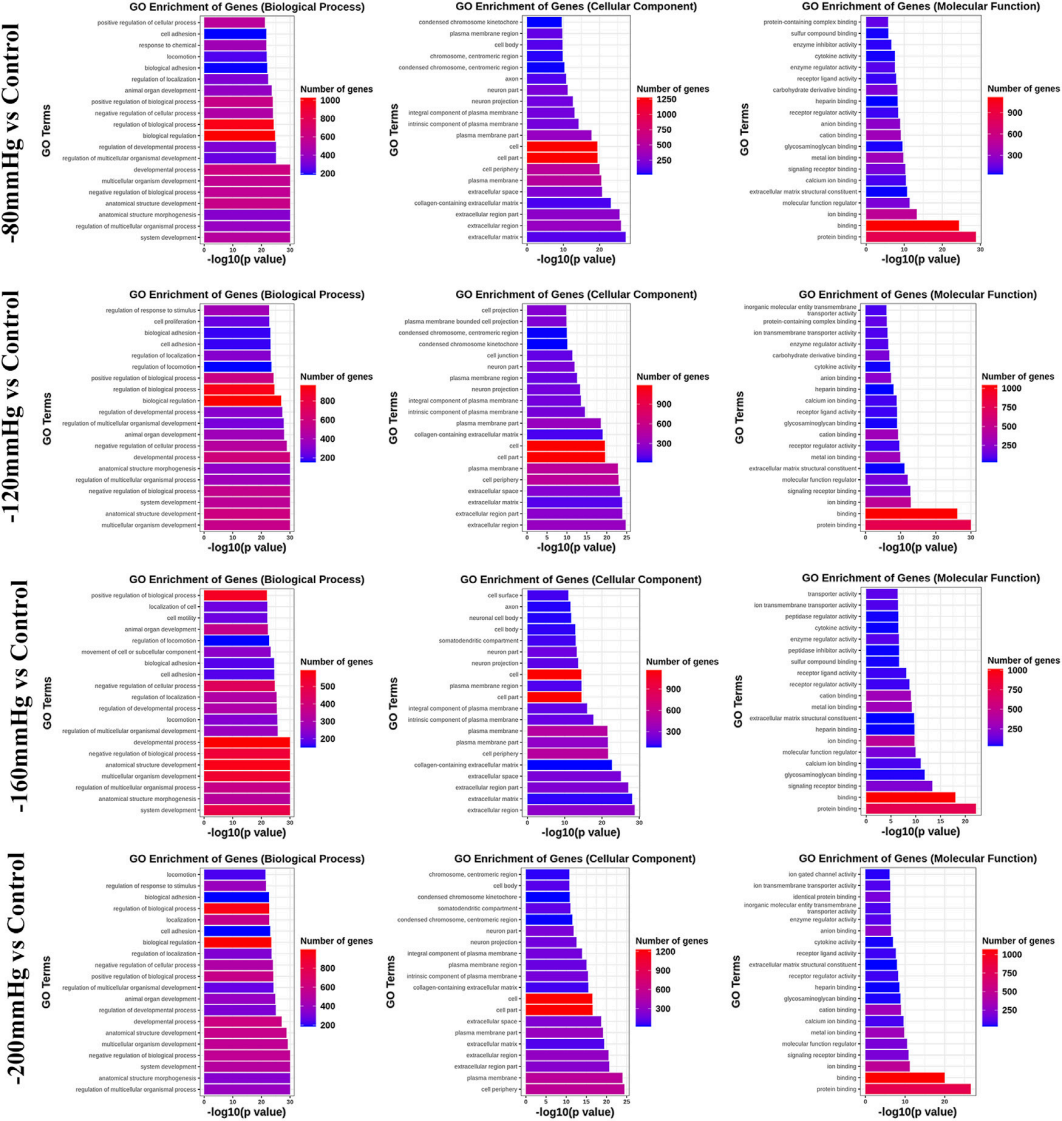
GO analysis of DEGs in −80 mmHg vs. control, −120 mmHg vs. control, −160 mmHg vs. control, and −200 mmHg vs. control.

**FIGURE 10:**
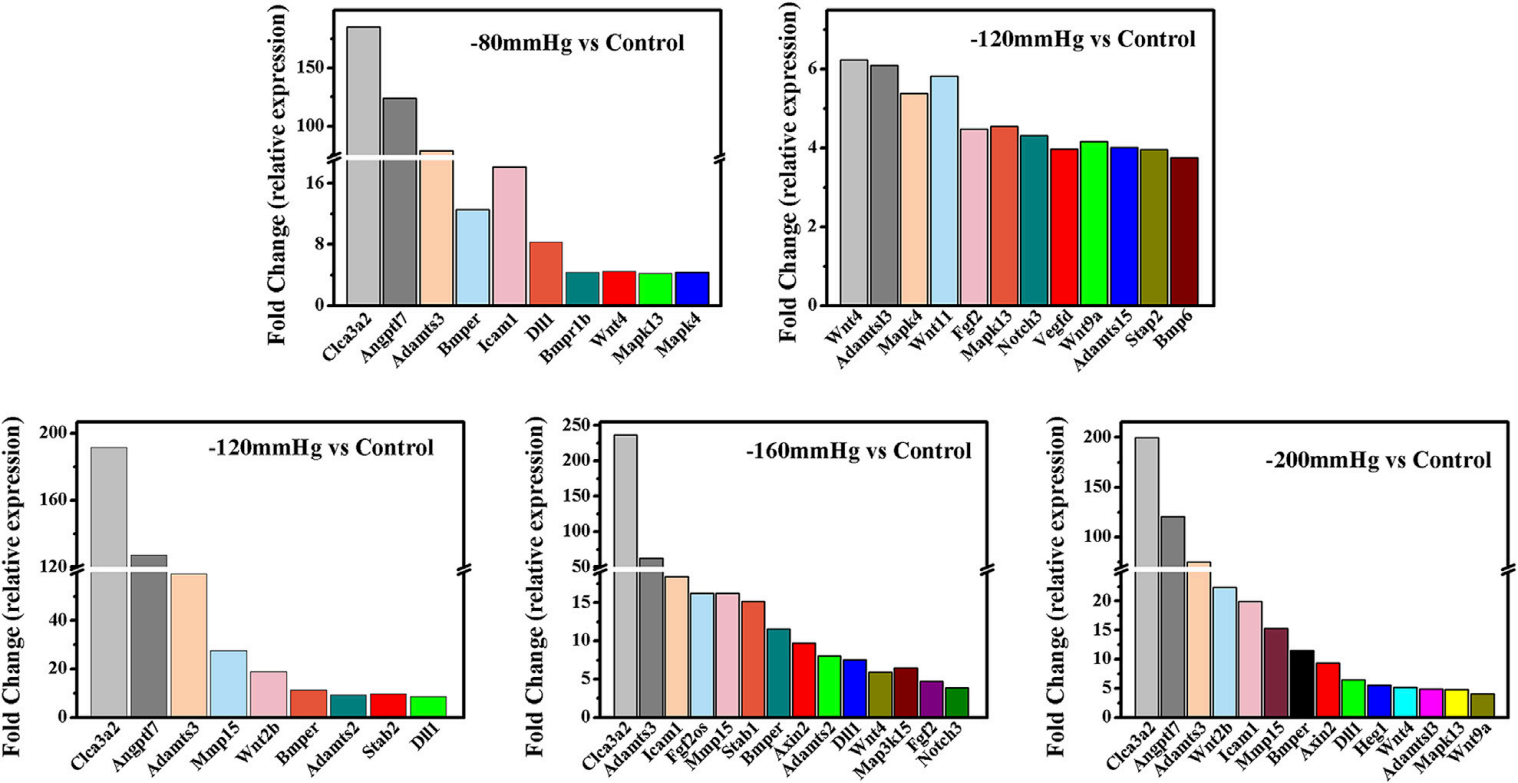
The osteogenesis associated DEGs in −80 mmHg vs. control, −120 mmHg vs. control, −160 mmHg vs. control, and −200 mmHg vs. control.

GO analysis (Figure 9) revealed significant enrichment of genes related to biological processes, molecular functions, and cellular components. The enriched items related to the osteogenic effects of the NP treatment were further analyzed. The enriched genes were assigned to the following categories: “regulation of response to stimulus,” “cell proliferation,” “collagen-containing extracellular matrix,” “extracellular matrix,” “calcium ion binding,” “extracellular matrix structural constituent,” and “signaling receptor binding.” Consistently, the upregulated expressions of osteogenesis-promoting genes, such as Clca3a2 (chloride channel accessory 3A2), Angptl7 (angiopoietin-related protein 7), Adamts3 (a disintegrin and metalloproteinase with thrombospondin motifs 3), BMP binding endothelial regulator (Bmper), Bmpr1b, Dll1, Wnt4, Mapk3, and Mapk4, were identified in the −80 mmHg treatment group. In the −120 mmHg treatment group, the upregulated expressions of osteogenesis-promoting genes were Clca3a2, Angptl7, Adamts3, Adamts15, Bmper, Bmp6, Dll1, Wnt4, Wnt11, Wnt2b, Wnt9a, Mmp15, Stab2, Stap2, Mapk13, Notch3, Vegfd, Fgf2, and Mapk4. Osteogenesis-associated DEGs in the −160 mmHg treatment group included Clca3a2, Adamts3, Icam1, Mmp15, Bmper, Axin2, Adamts2, Dll1, Map315k4, Wnt4, Fgf2, and Notch3. In the −200 mmHg treatment group, the osteogenesis-associated DEGs included Clca3a2, Angptl7, Adamts3, Wnt2b, Icam1, Mmp15, Bmper, Axin2, Dll1, Heg1, Wnt4, Adamts13, Mapk13, and Wnt9a (Figure 10).

**FIGURE 11:**
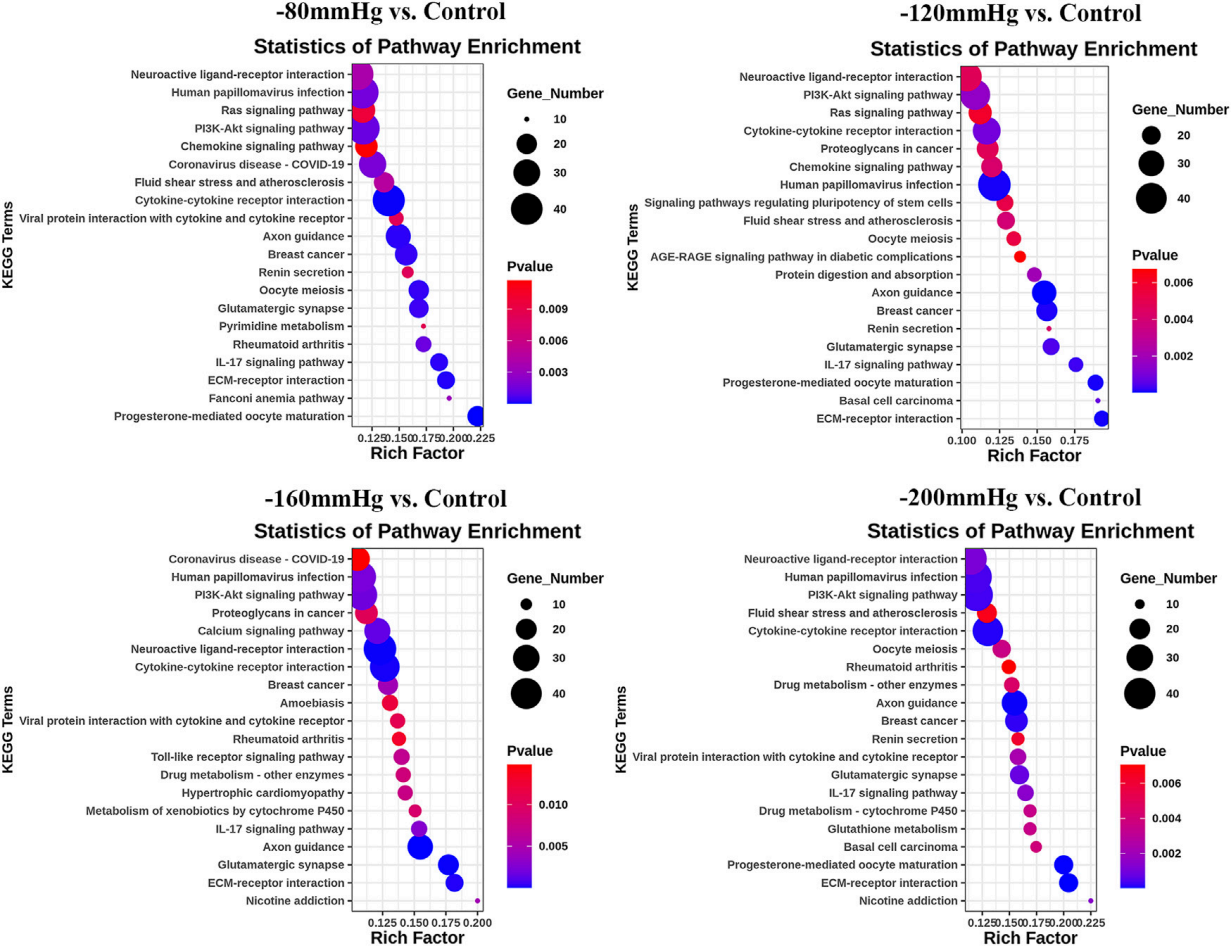
KEGG analysis of DEGs in −80 mmHg vs. control, −120 mmHg vs. control, −160 mmHg vs. control, and −200 mmHg vs. control.

**FIGURE 12:**
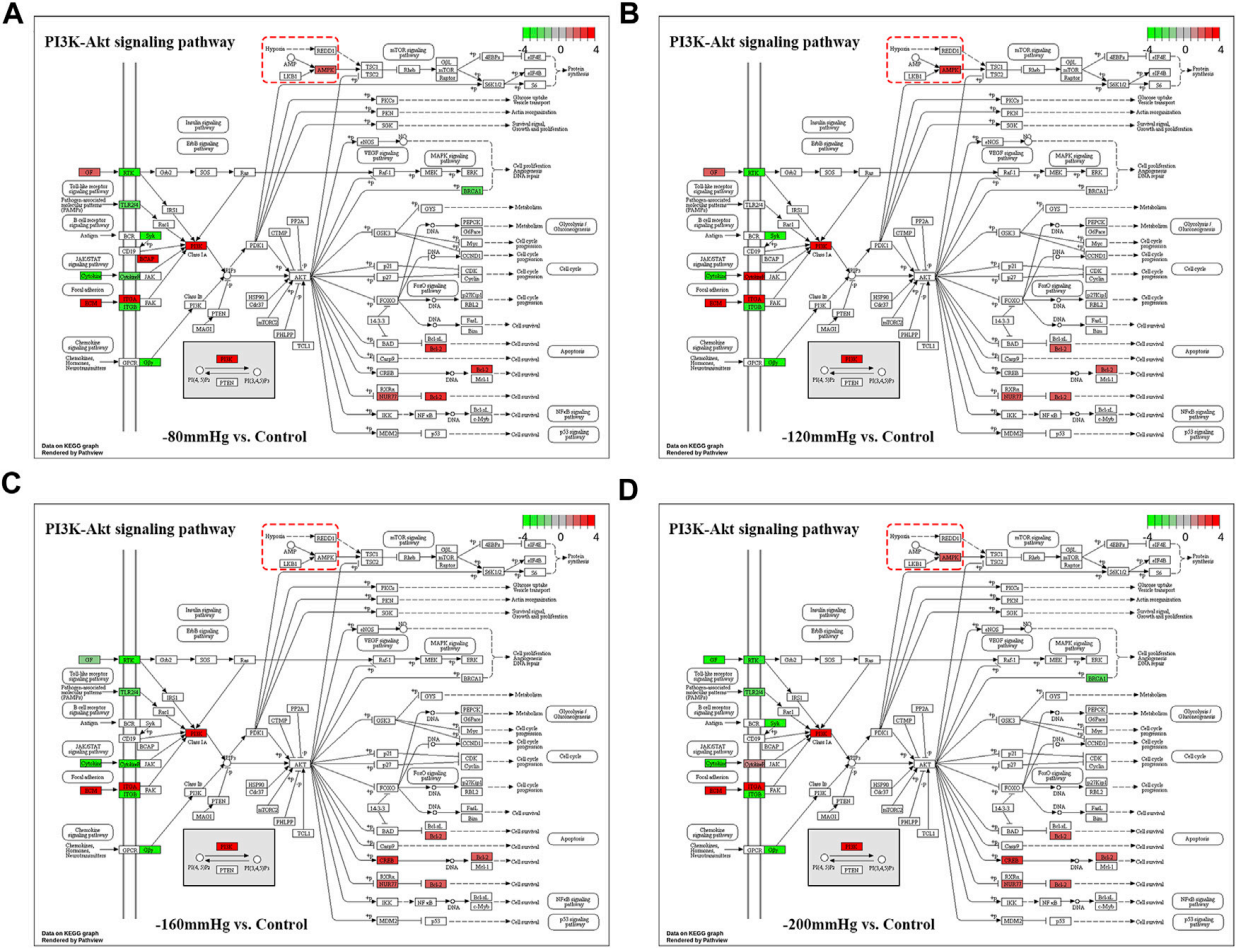
PI3K-Akt signaling pathway in −80mmHg vs. Control **(A)**, −120mmHg vs. Control **(B)**, −160mmHg vs. Control **(C)** and −200mmHg vs. Control **(D)**.

These results suggest that NP treatment systemically induced osteoblast differentiation in BMSCs, and the osteogenic effect was more evident in the −120 mmHg NP group. To explore the initial driver of the osteogenic effect of NP treatment, we performed KEGG enrichment analysis (Figure 11). We noticed that the “PI3K-Akt signaling pathway” as PI3K and Akt are the critical sensors of intracellular signal transduction pathways that promote metabolism, proliferation, cell survival, growth, and angiogenesis in response to extracellular signals. In PI3K-Akt signaling pathway, as showed in Figure 12, the DEGs enriched in the pathway activating molecules such as GF, ECM, ITGA, BCAP, PI3K, NUR77 and Bcl2, and thus activated PI3K-Akt signaling pathway.

**FIGURE 13:**
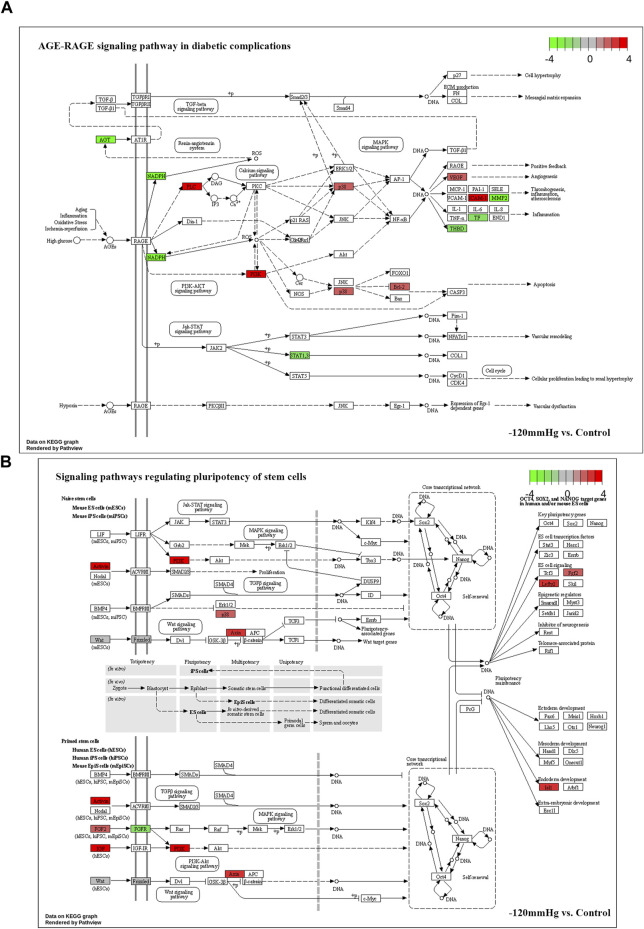
Potential activated signaling pathway under −120mmHg treatment. **(A)**AGE-RAGE signaling pathway in diabetic complications. **(B)** Signaling pathways regulating pluripotency of stem cells.

Noticeably, in −120mmHg vs. Control group, the molecules of AMPK and CytokineR were significantly activated, which may cause to be different from the others groups. In addition, as Figure 13 shows, PLC, PI3K, p38, Bcl-2, VEGF, ICAM-1 molecules were upregulated in AGE-RAGE signaling pathway in diabetic complications; and as Figure 13B demonstrates, the Activin, PI3K, p38, Axin, Fgf2, Lefty2, IGF, Isl1 molecules were upregulated in signaling pathways regulating pluripotency of stem cells. We suppose that osteoblast differentiation in BMSCs treated with −120 mmHg may also be regulated through “AGE-RAGE signaling pathway in diabetic complications” and “signaling pathways regulating pluripotency of stem cells”, which were not presented in the others NP scale.

### Proliferation and osteogenic differentiation of BMSCs

**FIGURE 14:**
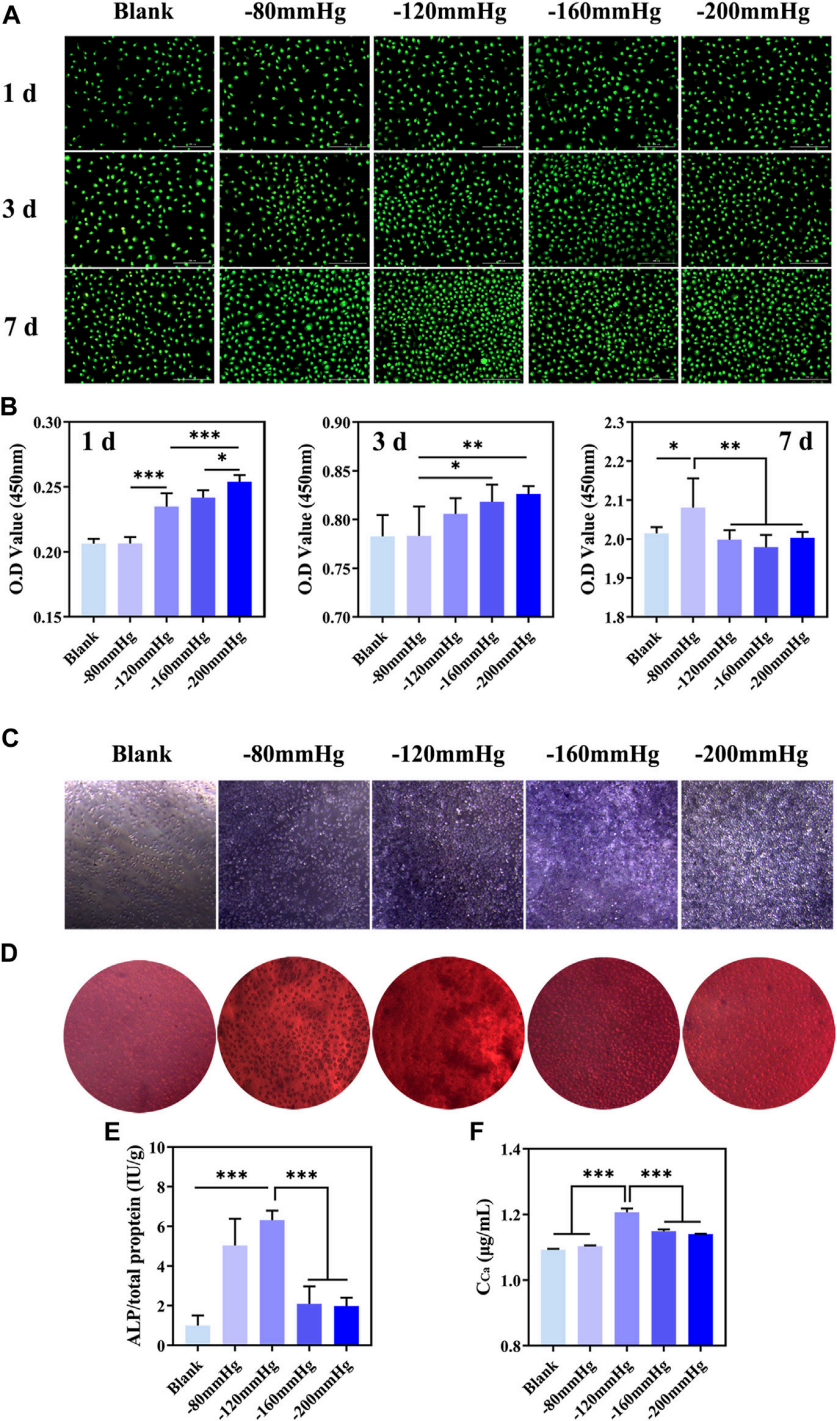
*In vitro* proliferation and osteogenic differentiation of BMSCs after NP treatment. **(A)** Live/dead staining of BMSCs at 1 d, 3 d and 7 d after NP treatment. **(B)** CCK-8 analysis of BMSCs at 1 d, 3 d and 7 d after NP treatment. **(C)** ALP staining of BMSCs at 7 d after NP treatment. **(D)** Alizarin red S staining of BMSCs at 12 d after NP treatment. **(E, F)** Quantification of ALP and calcium content of BMSCs.

The cell viability of BMSCs increased significantly after NP treatment, especially within 3 days (Figures 14A, B). ALP is an important marker in the early stage of osteoblastic differentiation. ALP staining showed that compared with NP of −80, −160 and −200 mmHg groups, the −120 mmHg had more ALP-positive cells, which suggested that osteogenic differentiation was promoted (Figure 14C). As shown in Figure 14E, ALP activity was maximal in −120 mmHg (6.315 ± 0.485 IU/g) compared with the −80 mmHg (5.037 ± 1.353 IU/g), −160 mmHg (2.096 ± 0.879 IU/g) and −200 mmHg (1.975 ± 0.427 IU/g). Calcium nodule formation is typically used as a marker to evaluate the later stage of cell osteogenesis. Consistently, more calcium nodules were observed in −120 mmHg group after 12 d of NP treatment (Figure 14D). The quantitative analysis of calcium nodules was shown in Figure 14F.

## Discussion

This study established a CFD model combining gene sequence analysis and osteogenic differentiation of BMSCs to investigate the working of NPWT on trabecular tissues, considering the effects of structural anisotropy and bone regeneration. The results showed the following: ①The pressure, shear stress on the trabeculae, and fluid velocity decreased exponentially as WD increased. ②The NP magnitude significantly affects the fluid dynamics, especially those close to the NP source. ③Anisotropy of trabecular structures coupled with anisotropic hydrodynamic behavior of the bone marrow. ④Trabecular regeneration leads to unsteady marrow flow induced by NP. ⑤−120 mmHg demonstrated the most osteogenic genes compared to −80 mmHg, − 160 mmHg, and 200 mmHg, indicating an optimal osteogenic effect under −120 mmHg treatment. The pressure, shear stress on trabeculae, and fluid velocity at any WD inside the marrow cavity can be theoretically quantified using the functional relation. These findings help improve understanding of the mechanisms underlying NPWT in trabecular tissue.

Bone tissue are abundant with blood supply. Bone structure, as well as the nourishing vessels, the lymphatic vessels, soft tissue are damaged when open fractures occur. Bone nourishing vessels and lymphangion connect to the circulatory system and thus fluid enters the bone marrow compartment (Beverly et al., 2018; Beverly and Murray, 2018; Chang et al., 2021; St-Pierre et al., 2022); furthermore, intraosseous pressure (pressure inside the bone marrow compartment) can up to tens of mmHg, allowing NPWT work. The results for shear stress (up to 100Pa) and velocity (up to 4 m/s) imply a flow rate of tens of liters per hour, which seems to reflect the unrealistic boundary conditions in this study, but there are two clinical scenarios need to be highlighted: ①In the case of large amounts of fluid (tens of milliliters) present in some severe fracture wounds, the calculated flow rate is reasonable but may only be valid for a short time because the amount of fluid is always limited and the broken capillaries, lymphatic vessels and damaged soft tissue (from which the fluid is coming) will gradually heal and close; ②When the substantial liquid was drained, or the liquid only exude at a low level, the vacuum pump can still generate negative pressure but there is not enough liquid to form a clear flow, resulting in a draw volume of a few milliliters per hour. Therefore, the fluid dynamics in this study should be considered only as the result under an ideal condition at the early stage of NP treatment.

Pressure gradients are fundamental causes of fluid motion, higher pressure gradients, and higher flow velocities. Our study showed that the pressure gradients increased significantly in the region close to the NP source, and the fluid flow velocity increased accordingly. According to Poiseuille’s law (Porret et al., 1998), fluid flow shear is proportional to the flow rate (which is positively correlated with flow velocity); therefore, the closer the NP source, the greater the shear stress of the fluid acting on the trabecular bone. It should be noted that the fluid domain size should match the trabecular specimen when developing such a fluid-solid CFD simulation (a 3 × 3 × 3 mm bone specimen was enclosed by a 3.4 × 3.4 × 3.4 mm fluid domain in this study). It is because the larger the fluid domain size, the smaller the pressure gradient within the trabecular bone and the less realistic the simulation results, provided the size of the trabecular specimen is constant. Anisotropy is one of the essential structural features of the trabecular bone. In terms of solid biomechanics, studies have confirmed that anisotropy effectively affects mechanical properties; one manifestation of this is that the strength of trabecular structures is significantly superior along the physiological axis than along the off-physiological axis (Maquer et al., 2015; Liu et al., 2019). In terms of fluid mechanics, similar patterns were observed in this study. When the NP was along the physiological axis (Z-axis), the flow velocity, shear stress, and pressure of each WD plane were, on average, higher than those of the NP off-physiological axis (along the non-Z axis). The fluid tends to be stable when the NP is along the physiological axis. It is not a coincidence that the bone marrow fluid behavior is similar to trabecular bone behavior in response to loading but is the result of fluid-solid coupling within the marrow cavity. Studies have shown that when the trabecular bone is under compressive loading, the flow direction of the bone marrow coincides with the physiological axis, and the trabecular structure reflects the loading pattern and the bone marrow fluid behavior (Metzger et al., 2015; Li et al., 2020; Rabiatul et al., 2021).

The essence of NP-induced bone marrow flow is its permeability. Porosity is a commonly used parameter for predicting the permeability of trabecular tissues (Teo and Teoh, 2012). When the porosity decreased, BV/TV increased accordingly. One could easily imagine an extremity where the fluid flow was finally blocked when BV/TV increased to nearly 100%. Therefore, the fluid flow tends to be sparse, and the pressure and shear stress upon the trabeculae decrease as bone regeneration proceeds (Figure 7). Yet, we cannot claim that changing the global threshold simulates complex biological processes of bone regeneration. In particular, the even/global structure regeneration could result in misinterpretations of the influence of trabecular repair on biomechanical behavior. Simulated bone regeneration offers an opportunity or tool to investigate phenomenological aspects of bone remodeling (Corrado et al., 2021; Han et al., 2022).

NPWT promotes angiogenesis and granulation in part by the strain-induced production of growth factors and cytokines, and most of these findings were recognized under an NP of −120 or −125 mmHg (Glass et al., 2014), which is most effective for soft-tissue wounds (Argenta and Morykwas, 1997; Morykwas et al., 1997; Morykwas et al., 2001; Chen et al., 2017; Liu et al., 2018b; Wu et al., 2022). In a bone tissue, which is a hard tissue, BMSCs osteogenesis activation and osteoclasts inhibition were also found under a short time continuous NP of −125 mmHg *via* kinds of osteogenic factors, such as vascular endothelial growth factor, bone morphogenetic protein-2, osteopontin, mechanotransduction molecule integrin β5, alkaline phosphatase, collagen1α2 and hypoxia-inducible factor-1α(Zhu et al., 2014; Wang et al., 2020; Zhu et al., 2021). The mechanisms by which NP activates mechanotransductive signaling pathways and stimulates the osteoblastic phenotype have not been elucidated. Our gene sequence analysis showed that the types of bone formation-related genes and signaling pathway (Figures 9–13) that have not been reported were identified in the −80 mmHg, −120 mmHg, −160 mmHg, and −200 mmHg groups, indicating that NP systemically promotes the differentiation of BMSCs into osteoblasts. However, the tendency was clear: −120 mmHg NP demonstrated more osteogenic genes. Further, we conducted the verification of proliferation and osteogenic differentiation of BMSCs, and the results confirm that −120 mmHg NP demonstrated the optimal osteogenic effect compare with other NP scales (Figure 14). This osteogenic differentiation was also verified by clinical practice and experimental findings (Kim and Lee, 2019; Zhu et al., 2021). One would argue that osteogenesis is a combined effect of fluid shear stress and NP within the marrow cavity; however, studies have shown that hydrodynamic shear stress induces prostaglandin synthesis, activation of kinases ERK and JNK, and mRNA expression of Runx2, ALP, COL1A1, VEGF, BMP-2, BMP -7 and TGF-β1, but does not stimulate cell proliferation in osteoblasts and MSCs (Kreke et al., 2008; Sharp et al., 2009; Yu et al., 2014).

To our knowledge, this is the first study to investigate bone marrow flow dynamics under NP. Combining the CFD results with gene sequence analysis and osteogenic differentiation of BMSCs, our findings are clinically informative. First, different NPs significantly changed the magnitude of pressure, shear stress on trabeculae, and fluid velocity only within a short WD; the suction effect tended to be marginal when the WD was deeper than 2.4 mm (Figure 5; Figure 7. Second, an NP of −120 mmHg may have optimal osteogenesis only within an effective WD. This trend suggests that we should not consider a higher NP to deal with open fractures or bone defects. Practice demonstrates that a high NP magnitude does not positively impact clinical outcomes and may even cause soft tissue complications (Normandin et al., 2021). Furthermore, according to the derived equations, the effective WD at a certain NP scale can be theoretically quantified. We suppose that effective WD should be such a condition that, as distance deepens, NP reaches a critical state that can no longer activate osteogenesis. However, the critical state remains to be further investigated.

Some limitations should be noticed. First, we did not consider soft tissue coverings, such as skin, fat, and muscle. Bones are covered by soft tissue, with only a few extreme cases in which the bones are entirely exposed and defective. NPWT decreases lateral tissue tension by 40%–50% while drawing wound edges closer (Wilkes et al., 2012; Loveluck et al., 2016). Limited NP suction on the bone due to obstruction from soft tissue deformation. Second, we only performed simulations in a single individual with a tiny trabecular cube, more samples with larger sizes are needed for further study. Third, the pattern of real trabecular bone remodeling remains unclear, and a bone remodeling simulation has not been carried out in present study. We can not claim that using a different lower threshold filter does actually simulate the complex biological processes of bone remodeling. Fourth, verification is still required. For example, a measuring sensor is placed in the marrow cavity to quantify the fluid dynamics induced by NP (Waldorff et al., 2007). Verification of the supposed signaling pathway in genes sequencing analysis should also be carried out. Fifth, intermittent suction protocols are used in clinical practice. Intermittent NP of −50 kPa (= −375 mmHg) has also been shown to activate kinds of molecules and proteins to induce BMSCs differentiation into bone cells (Zhang et al., 2010; Zhang et al., 2013). In addition, fluid flow features are induced by trabecular bone deformation under cyclic loading (Li et al., 2020), and fluid dynamics under intermittent protocols remain to be investigated.

## Conclusion

This study examined the marrow flow dynamics within trabecular bone under NPWT, and verified osteogenesis-related gene expression as well as osteogenic differentiation under the frequently used NP scale. The results showed that the magnitude and spatial distribution of pressure, shear stress, and fluid velocity varied with the NP direction and trabecular structure. Anisotropy of the trabecular structure coupled with anisotropic hydrodynamic behavior of the bone marrow. The pressure, shear stress on the trabeculae, and marrow fluid velocity decreased exponentially as WD increased. As WD deepens, the effect of the NP scale on pressure, shear stress, and fluid velocity become marginal.NP of −120 mmHg demonstrates most bone formation-related genes and the optimal *in vitro* osteogenic effect. Collectively, NP of −120 mmHg may have the optimal ability to promote osteogenesis, but the effective WD may be limited to a certain depth, which remains to be further investigated.

## Data Availability

The original contributions presented in the study are publicly available. This data can be found here: https://www.ncbi.nlm.nih.gov/geo/query/acc.cgi?acc=GSE216691
