# Supplementary material for: Combined computational analysis and cytology show limited depth osteogenic effect on bone defects in negative pressure wound therapy
Source: Front Bioeng Biotechnol. 2023 Feb 16;11:1056707. doi: 10.3389/fbioe.2023.1056707 (PMC9978480; doi:10.3389/fbioe.2023.1056707)
Supplement: Supplementary file 1 [file Table1.DOCX]

**Supplemental tables**

**Table S1**. Coefficients of exponential expression describing the dependent variables as a function of WD under an NP of −80 mmHg.

| NP direction | Dependent variable | A (P value) | B (P value) | R² |
| --- | --- | --- | --- | --- |
| along X | Pressure | -15.840(0.050) | -3.876(<0.001) | 0.985 |
|  | Velocity | 3.502(0.006) | -1.854(<0.001) | 0.978 |
|  | Shear stress | 104.152(0.001) | -2.347(<0.001) | 0.993 |
| along Y | Pressure | -19.067(0.006) | -3.567(<0.001) | 0.994 |
|  | Velocity | 4.604(0.001) | -1.925(<0.001) | 0.991 |
|  | Shear stress | 82.727(0.012) | -1.974(<0.001) | 0.973 |
| along Z | Pressure | -8.181(0.002) | -2.225(<0.001) | 0.991 |
|  | Velocity | 2.987(0.001) | -1.167(<0.001) | 0.974 |
|  | Shear stress | 68.731(<0.001) | -1.227(<0.001) | 0.985 |

**Table S2**. Coefficients of exponential expression describing the dependent variables as a function of WD under an NP of −120 mmHg.

| NP direction | Dependent variable | A (P value) | B (P value) | R² |
| --- | --- | --- | --- | --- |
| along X | Pressure | -22.528(0.052) | -3.793(<0.001) | 0.983 |
|  | Velocity | 4.183(0.007) | -1.828(<0.001) | 0.977 |
|  | Shear stress | 123.045(0.002) | -2.281(<0.001) | 0.992 |
| along Y | Pressure | -28.495(0.006) | -3.566(<0.001) | 0.994 |
|  | Velocity | 5.588(0.001) | -1.918(<0.001) | 0.991 |
|  | Shear stress | 101.339(0.012) | -1.945(<0.001) | 0.972 |
| along Z | Pressure | -12.226(0.002) | -2.222(<0.001) | 0.990 |
|  | Velocity | 3.642(0.001) | -1.164(<0.001) | 0.973 |
|  | Shear stress | 83.819(<0.001) | -1.207(<0.001) | 0.984 |

**Table S3**. Coefficients of exponential expression describing the dependent variables as a function of WD under an NP of −160 mmHg.

| NP direction | Dependent variable | A (P value) | B (P value) | R² |
| --- | --- | --- | --- | --- |
| along X | Pressure | -30.489(0.052) | -3.825(<0.001) | 0.984 |
|  | Velocity | 4.902(0.007) | -1.845(<0.001) | 0.977 |
|  | Shear stress | 141.365(0.002) | -2.276(<0.001) | 0.992 |
| along Y | Pressure | -38.532(0.007) | -3.582(<0.001) | 0.994 |
|  | Velocity | 6.499(0.001) | -1.923(<0.001) | 0.992 |
|  | Shear stress | 116.669(0.014) | -1.929(<0.001) | 0.970 |
| along Z | Pressure | -16.232(0.002) | -2.221(<0.001) | 0.990 |
|  | Velocity | 4.183(0.001) | -1.158(<0.001) | 0.972 |
|  | Shear stress | 96.247(<0.001) | -1.194(<0.001) | 0.983 |

**Table S4**. Coefficients of exponential expression describing the dependent variables as a function of WD under an NP of −200 mmHg.

| NP direction | Dependent variable | A (P value) | B (P value) | R² |
| --- | --- | --- | --- | --- |
| along X | Pressure | -38.352(0.051) | -3.839(<0.001) | 0.984 |
|  | Velocity | 5.427(0.007) | -1.836(<0.001) | 0.976 |
|  | Shear stress | 157.173(0.002) | -2.253(<0.001) | 0.992 |
| along Y | Pressure | -46.698(0.007) | -3.577(<0.001) | 0.994 |
|  | Velocity | 7.192(0.001) | -1.923(<0.001) | 0.991 |
|  | Shear stress | 127.665(0.015) | -1.911(<0.001) | 0.969 |
| along Z | Pressure | -20.376(0.002) | -2.221(<0.001) | 0.990 |
|  | Velocity | 4.695(0.001) | -1.161(<0.001) | 0.973 |
|  | Shear stress | 107.675(<0.001) | -1.185(<0.001) | 0.983 |

**Table S5**. Dependent variable as an exponential function of WD at the BV/TV of 13.07% under −80 mmHg.

| NP direction | Dependent variable | A (P value) | B (P value) | R² |
| --- | --- | --- | --- | --- |
| along X | Pressure | -14.383(<0.001) | -3.072(<0.001) | 0.999 |
|  | Velocity | 4.389(<0.001) | -1.506(<0.001) | 0.998 |
|  | Shear stress | 69.851(<0.001) | -1.543(<0.001) | 0.991 |
| along Y | Pressure | -13.566(<0.001) | -2.911(<0.001) | 0.998 |
|  | Velocity | 4.612(<0.001) | -1.502(<0.001) | 0.999 |
|  | Shear stress | 79.943(<0.001) | -1.574(<0.001) | 0.994 |
| along Z | Pressure | -10.684(<0.001) | -2.543(<0.001) | 0.999 |
|  | Velocity | 4.061(<0.001) | -1.283(<0.001) | 0.999 |
|  | Shear stress | 76.962(<0.001) | -1.346(<0.001) | 0.997 |

**Table S6**. Dependent variable as an exponential function of WD at the BV/TV of 18.85% under –80 mmHg.

| NP direction | Dependent variable | A (P value) | B (P value) | R² |
| --- | --- | --- | --- | --- |
| along X | Pressure | -16.283(0.002) | -3.317(<0.001) | 0.996 |
|  | Velocity | 4.562(<0.001) | -1.643(<0.001) | 0.996 |
|  | Shear stress | 84.442(0.001) | -1.810(<0.001) | 0.992 |
| along Y | Pressure | -15.690(<0.001) | -3.109(<0.001) | 0.998 |
|  | Velocity | 4.856(<0.001) | -1.618(<0.001) | 0.999 |
|  | Shear stress | 89.606(0.003) | -1.695(<0.001) | 0.981 |
| along Z | Pressure | -9.931(<0.001) | -2.423(<0.001) | 0.998 |
|  | Velocity | 3.7521(<0.001) | -1.223(<0.001) | 0.996 |
|  | Shear stress | 84.595(<0.001) | -1.363(<0.001) | 0.993 |

**Table S7**. Dependent variable as an exponential function of WD at the BV/TV of 24.97% under −80 mmHg.

| NP direction | Dependent variable | A (P value) | B (P value) | R² |
| --- | --- | --- | --- | --- |
| along X | Pressure | -16.017(0.006) | -3.498(<0.001) | 0.994 |
|  | Velocity | 3.757(0.001) | -1.681(<0.001) | 0.988 |
|  | Shear stress | 89.638(0.002) | -2.030(<0.001) | 0.990 |
| along Y | Pressure | -18.505(0.002) | -3.312(<0.001) | 0.996 |
|  | Velocity | 4.723(<0.001) | -1.755(<0.001) | 0.998 |
|  | Shear stress | 88.449(0.011) | -1.848(<0.001) | 0.971 |
| along Z | Pressure | -8.315(<0.001) | -2.240(<0.001) | 0.996 |
|  | Velocity | 3.118(<0.001) | -1.153(<0.001) | 0.986 |
|  | Shear stress | 71.436(<0.001) | -1.256(<0.001) | 0.988 |

**Table S8**. Dependent variable as an exponential function of WD at the BV/TV of 31.94% under −80 mmHg.

| NP direction | Dependent variable | A (P value) | B (P value) | R² |
| --- | --- | --- | --- | --- |
| along X | Pressure | -15.870(0.050) | -3.878(<0.001) | 0.985 |
|  | Velocity | 3.502(0.006) | -1.854(<0.001) | 0.978 |
|  | Shear stress | 104.152(0.001) | -2.347(<0.001) | 0.993 |
| along Y | Pressure | -19.086(0.006) | -3.568(<0.001) | 0.994 |
|  | Velocity | 4.604(0.001) | -1.925(<0.001) | 0.991 |
|  | Shear stress | 82.727(0.012) | -1.974(<0.001) | 0.973 |
| along Z | Pressure | -8.181(0.002) | -2.225(<0.001) | 0.991 |
|  | Velocity | 2.987(0.001) | -1.167(<0.001) | 0.974 |
|  | Shear stress | 68.731(<0.001) | -1.227(<0.001) | 0.985 |
